# Evolution and genetics of bighead and silver carps: Native population conservation versus invasive species control

**DOI:** 10.1111/eva.12982

**Published:** 2020-05-13

**Authors:** Guoqing Lu, Chenghui Wang, Jinliang Zhao, Xiaolin Liao, Jun Wang, Mingkun Luo, Lifeng Zhu, Louis Bernatzhez, Sifa Li

**Affiliations:** ^1^ Department of Biology University of Nebraska at Omaha Omaha NE USA; ^2^ Key Laboratory of Aquatic Genetic Resources and Aquacultural Ecosystems Ministry of Agriculture Shanghai Ocean University Shanghai China; ^3^ Key Laboratory of Exploration and Utilization of Aquatic Genetic Resources Ministry of Education Shanghai Ocean University Shanghai China; ^4^ Institute of Hydroecology Ministry of Water Resources & Chinese Academy of Sciences Wuhan China; ^5^ Wuxi Fisheries College Nanjing Agricultural University Jiangsu, Wuxi China; ^6^ College of Life of Sciences Nanjing Normal University Nanjing China; ^7^ IBIS (Institut de Biologie Intégrative et des Systèmes) Université Laval Québec QC Canada

**Keywords:** bigheaded carps, evolution, invasive species control, native population conservation, natural hybridization, population genetics

## Abstract

Bighead carp (*Hypophthalmichthys nobilis*) and silver carp (*H. molitrix)*, collectively called bigheaded carps, are cyprinids native mainly to China and have been introduced to over 70 countries. Paleontological and molecular phylogenetic analyses demonstrate bighead and silver carps originated from the Yangtze‐Huanghe River basins and modern populations may have derived from the secondary contact of geographically isolated fish during the last glacial events. Significant genetic differences are found among populations of native rivers (Yangtze, Pearl, and Amur) as well as introduced/invasive environments (Mississippi R., USA and Danube R., Hungary), suggesting genetic backgrounds and ecological selection may play a role in population differentiation. Population divergence of bighead carp or silver carp has occurred within their native rivers, whereas, within the Mississippi River Basin (MRB)—an introduced region, such genetic differentiation is likely taking place at least in silver carp. Interspecific hybridization between silver and bighead carps is rare within their native regions; however, extensive hybridization is observed in the MRB, which could be contributed by a shift to a more homogenous environment that lacks reproductive isolation barriers for the restriction of gene flow between species. The wild populations of native bighead and silver carps have experienced dramatic declines; in contrast, the introduced bigheaded carps overpopulate the MRB and are considered two invasive species, which strongly suggests fishing capacity (overfishing and underfishing) be a decisive factor for fishery resource exploitation and management. This review provides not only a global perspective of evolutionary history and population divergence of bigheaded carps but also a forum that calls for international research collaborations to deal with critical issues related to native population conservation and invasive species control.

## 1 INTRODUCTION

The bighead carp (*Hypophthalmichthys nobilis* or *Aristichthys nobilis*) and silver carp (*H. molitrix*), collectively called bigheaded carps or Chinese carp, belong to the family Cyprinidae, the largest family of freshwater fishes (Nelson, Grande, & Wilson, 2016). Both carps are characterized by stout bodies, large heads, and massive opercles (gill covers) (Kolar et al., 2007) (Figure 2). The bighead carp exhibits long, thin thread‐like gill rakers that are adapted for zooplankton feeding whereas the silver carp possesses sponge‐like gill rakers for phytoplankton filtering (Figure 2). The native range of silver carp extends from approximately 20°N to 54°N, including the areas from the Red River (Northern Vietnam), Zhujiang (Pearl) River (southern China) and north to the Heilongjiang (Amur) River (the China—Russia border). The bighead carp's native range is more narrow, from approximately 21°N to 40°N, covering the Zhujiang River north to the Huanghe (Yellow) River (northern China), but not to the Heilongjiang River (Li & Fang, 1990; Li, Wu, Wang, Chou, & Chen, 1990; Lu, Li, & Bernatchez, 1997).

**FIGURE 1:**
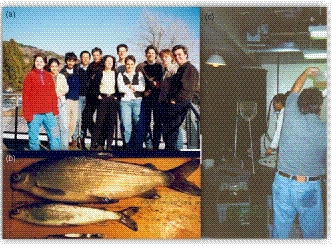
Memorable times in the Bernatchez Lab at Laval. (a) Laboratory crews went out occasionally for socials *circa* 1998; (b) Dwarf and normal lake whitefish, the iconic fish in the lab; (c) Louis was examining fertilized eggs in a cross experiment with dwarf and normal lake whitefish while I was preparing for hatching equipment

**FIGURE 2:**
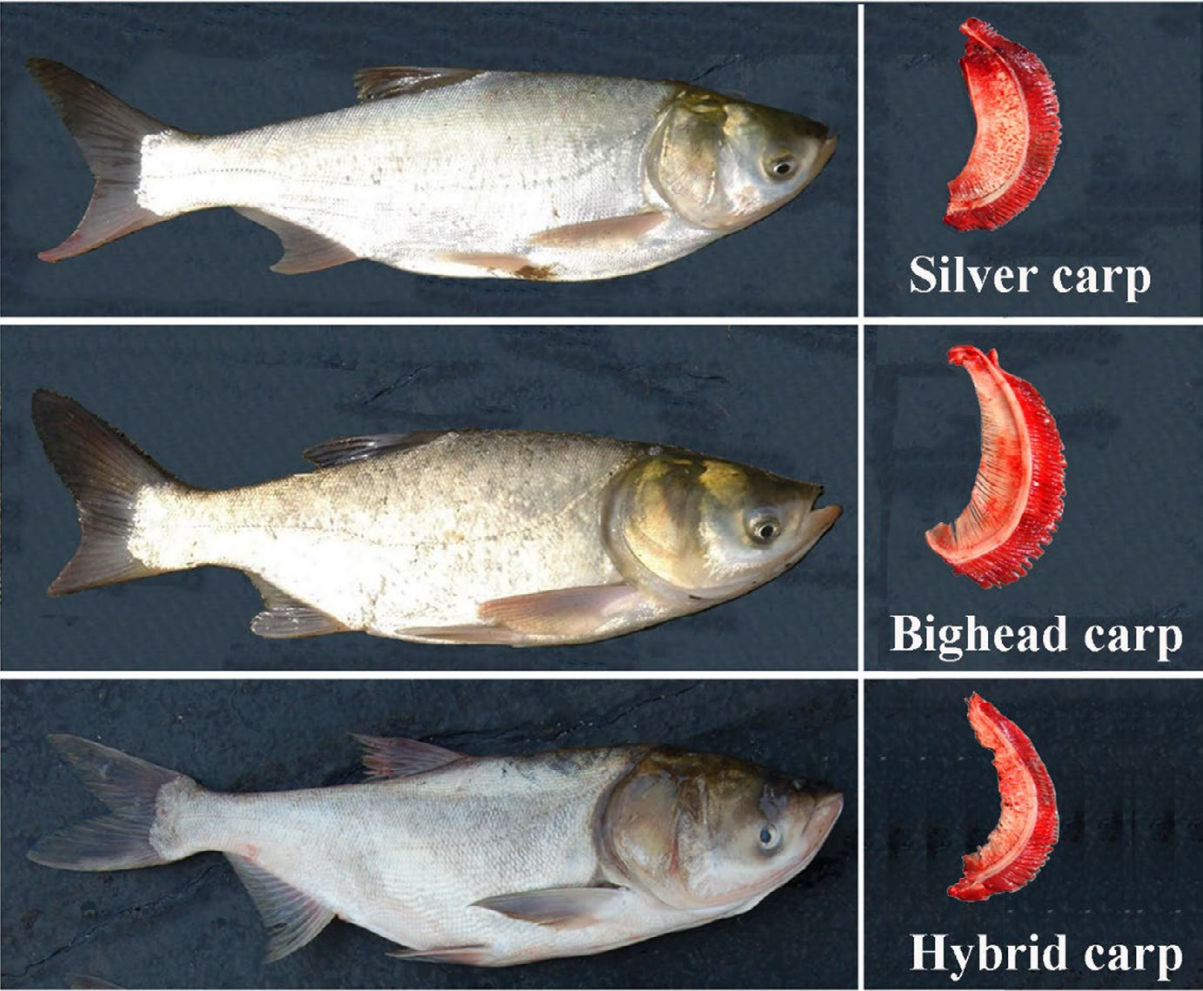
Bighead carp, silver carp and a hybrid with their corresponding gill rakers illustrated. Images of hybrid fish and gill rakers by courtesy of Duane Chapman (Columbia Environmental Research Center, USGS)

BOX 1 **Personal Reflections**

I (Lu) am very fortunate that I have met many great scientists. Among them, Prof. Sifa Li and Dr. Louis Bernatchez impact me most in my academic career and personal life. Prof. Li at the Shanghai Ocean University (SHOU) was my master's thesis advisor and offered me a research assistant position following my graduation. He was one of the earliest investigators who applied allozyme electrophoresis to study aquaculture genetics and a founder in fish germplasm research and conservation in China. In the 1980s, Prof. Li and his collaborators revealed significant morphological and genetic differences among populations of three large rivers, that is, Yangtze, Pearl and Amur in bighead, silver and grass carps (Li et al., 1990). Along this research line, Prof. Li proposed to study the population genetic structure of these Chinese carps within the Yangtze River, with funding support from *The International Development Research Centre *(IDRC), Canada. In the early 1990s, Louis had already been known for his pioneering studies on the evolutionary origin of lake whitefish using mtDNA‐RFLP (Bernatchez & Dodson, 1990). Prof. Li decided to have me visit the Bernatchez Lab at Laval University (Figure 1a) where I learned, for the first time, different molecular techniques and applied them to analyze population samples of four major Chinese carps collected from the Yangtze River. We published our first collaborative paper, which had an influence over the use of mtDNA‐RFLP in population genetic studies in China and offered a guideline for policies made to preserve the genetic resources of four major Chinese carps in the Yangtze River (Lu et al., 1997). This training also equipped me with molecular skills that allowed setting an mtDNA‐RFLP facility at SHOU.

I joined the Bernatchez Lab again in 1996 as a PhD student. My doctoral thesis focused on the understanding of evolutionary processes and speciation mechanisms of lake whitefish (Figure 1b) in eastern North America, which is an extension of Louis’ original work (Bernatchez & Dodson, 1990). To investigate evolutionary processes involved in the formation of sympatric lake whitefish, we performed a combined analysis of mitochondrial DNA and microsatellite loci among 36 populations and found that both scenarios of sympatric and allopatric origins might have occurred in dwarf and normal ecotypes depending on the lake (Lu et al., 2001). When comparing morphological differentiation and genetic divergence between sympatric dwarf and normal ecotypes from six lakes of the St. John River basin, we observed a negative correlation between gene flow and morphological differences in trophic‐related traits, which supports the ecological speciation hypothesis in sympatric lake whitefish (Lu & Bernatchez, 1999a).

To investigate possible reproductive isolation mechanisms in sympatric lake whitefish, we conducted cross experiments between dwarf and normal ecotypes and found daily embryonic mortality rates were higher in reciprocal hybrid crosses compared to pure crosses (Figure 1c; Lu & Bernatchez, 1998). We compared fluctuating asymmetry in morphological traits of the same hybrid and pure crosses and found that genetic stress observed in hybrids at embryonic stages was not manifested at later developmental stages (Lu & Bernatchez, 1999b). A more comprehensive understanding of adaptive divergence and reproductive isolation in sympatric lake whitefish has been achieved in succeeding studies that employed quantitative trait mapping, high‐throughput sequencing and other approaches (Bernatchez et al., 2010; Duan et al., 2009).

Regarding my career development, I took a different path rather than becoming a postdoctoral fellow after receiving a doctoral degree in Biology. I studied for a graduate certificate in Computer Science and envisioned an interdisciplinary career. I am very grateful that I can speak both Biology and Computer Science languages and find my passion in bioinformatics. In collaboration with many excellent investigators in the U.S. and other countries, my laboratory has developed a number of bioinformatics algorithms and databases used for data analysis and knowledge mining in biology and biomedicine while maintaining a relatively active research program in Asian carp (Wang, Lamer, et al., 2016; Wang, Gaughan, et al., 2019). Many of Louis's characters, including his love for and dedication to science and nature, his sincerity and care for students, and his optimistic attitude and diligence, have influenced numerous students, postdocs and visiting scholars who worked directly or indirectly in the Bernatchez Lab.

The bigheaded carps are major aquaculture species in East Asian and some European countries and ranked second (silver carp) and fifth (bighead carp) with fish production in world aquaculture in 2016 (FAO, 2018). Cultivation of bigheaded carps and other Chinese carps, for example, grass carp and black carp, has a long history that can be dated back to the Tang dynasty (618–907 AD) (Li et al., 1990). The carps are large semi‐migratory fishes in large rivers and lakes, grow and mature in floodplain lakes, and spawn in large rivers (often triggered by increased temperature and rising water levels) in the wild (Li et al., 1990). The hatching of fertilized eggs and the development of larvae occur during the downstream drift. Juvenile fish subsequently enter nursery areas and grow up in the floodplain lakes (Hu, Hua, Zhou, Wu, & Wu, 2015). Due to reasons such as overfishing, water pollution, habitat loss, and hydroelectric facilities (e.g., the Three Gorges Dam), the natural resources of theses Chinese carps in large rivers have declined considerably during the past decades (Li et al., 1990).

The bigheaded carps were introduced to over 70 countries and territories (Kolar et al., 2007). The reasons for introductions include aquaculture, capture fisheries enhancement, and the control of plankton and aquatic weeds (Froese & Pauly, 2000; Kolar et al., 2007). In certain introduced environments, bigheaded carps outcompete indigenous species and become extremely abundant (Fuller, Nico, & Williams, 1999; Laird & Page, 1996). In North America, the bigheaded carps were considered two highly undesirable invasive species (Conover, Simmonds, & Whalen, 2007; Irons, Sass, McClelland, & Stafford, 2007) and federal and local government agencies have been making enormous efforts to prevent them from spreading into the Laurentian Great Lakes, which could potentially damage an annual $7 billion fishing industry (Chapman, Chen, & Hoover, 2016).

In this paper, we describe the major findings of a long‐term research program on the conservation and utilization of genetic resources of Chinese carps (Box 1) and provide an update of knowledge concerning the evolutionary history and population genetics of bigheaded carps. Addressing the significance of native population conservation and invasion control, we review the current status of bigheaded carps in native and invasive environments and discuss possible strategies to address the management challenges. We also discuss the evolutionary consequences of interspecific hybridization that occurs extensively in invasive areas and provide our perspectives on future research directions.

## 2 TAXONOMIC CLASSIFICATION OF BIGHEADED CARPS

The taxonomic placement of bighead and silver carps species has been less consistent at the generic level, which occasionally causes confusion (Howes, 1981; Oshima, 1919). Bighead and silver carps were originally described as species of the genus *Leuciscus* and subsequently placed in the genus *Hypophthalmichthys*. Oshima (1919) reclassified the bighead carp to the genus *Aristichthys*. Distinct morphological characters such as the gill raker morphology, the position of the abdominal keel, and the pharyngeal dentition have led Chinese ichthyologists to classify bighead and silver carps into difference genera, that is, *Aristichthys* and *Hypophthalmichthys*. However, many western ichthyologists placed them in the same genus *Hypophthalmichthys* (Howes, 1981; Nelson et al., 2016). The comparative analysis of mitochondrial genomes of intergeneric cyprinid species found the genetic distance between bighead and silver carps below the smallest distance between intergeneric species, but within the range of pairwise genetic distances between intrageneric species, which supports the classification of bighead and silver carps as belonging to the same genus, that is, *Hypophthalmichthys* (Li et al., 2009).

The bigheaded carps are believed to have evolved from primitive cyprinids in central China during the late Neogene period, ~2.58–23.03 MYA (Li & Fang, 1990; Tao et al., 2010; Wang, Gaughan, et al., 2019). Fossil records of bigheaded carps were discovered in deposits of various epochs, such as the Pliocene (Yushe Basin, Shanxi, China), Pleistocene (Linfen, Shanxian, and Sanmenxia, Henan, China), and Holocene (Yichang, Hubei, China) (Li, 1981). The world's oldest bighead carp (37 MA) has been predicted based on excavations of the open lignite‐mining pit Na Duong in Vietnam (Böhme, Aiglstorfer, & Antoine, 2013). Whether bighead carp is an ancestral species in *Hypophthalmichthys* remains to be resolved. The analysis of a supergene matrix (100 genes) estimated the divergence between bighead and silver carps occurred ∼ 3.41 MYA (Tao et al., 2010). We recently conducted a phylogenomic analysis of 12 ray‐finned fishes based upon 950 single copy orthologues genes and found that the bighead and silver carps likely diverged much earlier, ~9.58 MYA (Wang, Gaughan, et al., 2019).

**FIGURE 3:**
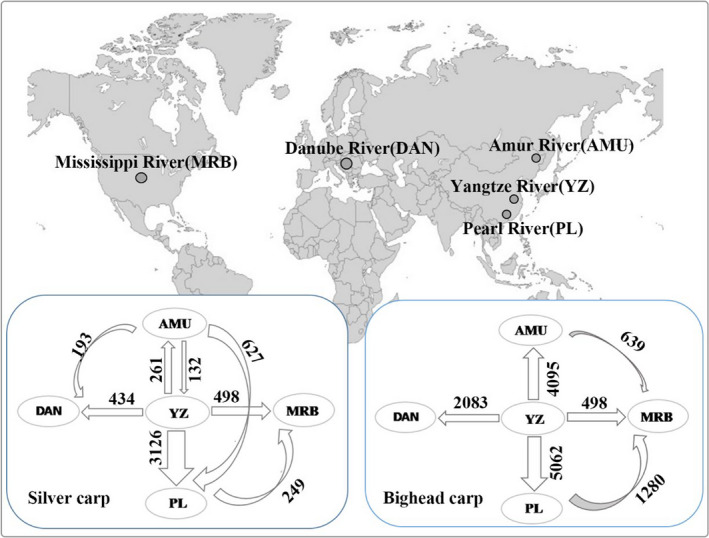
Sampling sites of bigheaded carps and gene flow between populations. MtDNA sequence data were from (Li et al., 2010, 2011) and gene flow was estimated with Migrate‐2.4.3 (Beerli & Felsenstein, 2001). The numbers indicate the magnitude of gene flow and the arrows indicate the direction of gene flow

Paleontological data indicate approximately 110 KYA during the Pleistocene, silver carp might have arrived in the Amur River through the Liaohe River and reached the Pearl River through the Yangtze River and the Qiantang River (Li et al., 1990). The native range of bighead carp is mainly the Yangtze River and the Pearl River watersheds. It was introduced to other river systems in China, including the Amur River (Li et al., 1990; Liu & He, 1992). Historically, the bigheaded carps are abundant in natural fish production in the Yangtze River (Li et al., 1990). The analysis of mitochondrial DNA sequences provided additional evidence to support the hypothesis that bigheaded carps originated from the Yangtze River basin (Figure 3) (Li et al., 2010, 2011). In both carps, the native Amur and Pearl River populations shared haplotypes with the Yangtze population but not with each other (Li et al., 2010, 2011).

Within the Yangtze River, two mtDNA haplotype clusters were revealed in bighead carps (Li et al., 2010, 2011; Lu et al., 1997). An analogous pattern was found in the Yangtze River grass carp (Lu et al., 1997; Zhao et al., 2011). The two phylogenetically distinct lineages of bigheaded carps may be attributed to the secondary contact of geographically isolated populations during the glacial events (Zhao et al., 2011). Geologically, the upstream portion of the Yangtze River belonged to a different paleo‐drainage network compared to the middle and downstream portions and was separated by the Three Gorges area; the connection of the Yangtze River at the Three Gorges was completed by ~ 2.58 MYA (Li et al., 1990). It is thus likely that the two distinct lineages were formed during this prior geographic isolation. This hypothesis should be validated by further studies with more species of fishes in the Yangtze River Basin.

## 3 GENETIC VARIATION AND DIVERSITY OF BIGHEADED CARPS

The bigheaded carps exhibited significant genetic differentiation among populations in their native regions (Table 1) (Li et al., 1990; Lu et al., 1997). Population genetic study of bigheaded carps in large river basins in China, pioneered by S. Li and his research team at the Shanghai Ocean University, found significant biochemical genetic variation in allozyme loci among the Yangtze, Pearl and Amur rivers (Li, 1986). Li and his team also observed the three populations of bigheaded carps possessed significant differences in morphometric and meristic characters (Li, Zhou, Ni, & Chen, 1989). Subsequent studies included samples not only from the Yangtze, Pearl and Amur Rivers (native) but also the Danube River, Hungary and the Mississippi River Basin (MRB), USA (introduced or invasive), identified significant genetic differences among native and invasive populations using multiple molecular techniques, including DNA sequencing (Li et al., 2010, 2011), AFLP (Yan, Zhao, Li, & Cao, 2011; Yan, Zhoa, Li, & Cheng, 2010) and microsatellites (Chen et al., 2012)*.* We analyzed the RAD‐Seq data kindly shared by J. Lamer (Illinois Natural History Survey) and found significant genetic differentiation between the Yangtze and Mississippi populations in both bighead and silver carps. This genetic differentiation between native and introduced populations might be explained by the variations in genetic backgrounds, genetic drift, and ecological selection (Lamer, Dolan, Petersen, Chick, & Epifanio, 2010).

**TABLE 1:** Genetic differentiation among native and introduced/invasive populations in bighead carp and silver carp

	Fst (mtDNA) / Nei's Distance (AFLP)/Fst (microsatellites)			
	Yangtze	Pearl	Amur	Danube
Silver carp				
Pearl	0.006^NS^/0.993*/0.181			
Amur	0.072**/0.993*/0.117	0.064 ^NS^/0.992*/0.172		
Danube	0.014 ^NS^/0.994*	0.118*/0.993*/?	0.100*/0.992*/?	
MRB	0.326**/0.991* 0.202–0.356	0.395** 0.991*/0.204–0.356	0.495 **/0.991*/0.244–0.387	0.478**/0.992* /?
Bighead carp				
Pearl	0.286**/0.21*/0.257			
Amur	0.033^NS^/0.425*/?	0.142**/0.29*/?		
Danube	0.164**/0.422*	0.436**/0.182*	0.1947**/0.338*	
MRB	0.226**/0.399*/0.208–0.257	0.164**/0.145*/0.298–0.406	0.139*/0.332*	0.315**/0.175* /?

Note:

Data from (Farrington et al. 2017; Li et al. 2011; Li et al. 2010; Yan et al. 2011; Yan et al. 2010)

Abbreviation: MRB, Mississippi River Basin, NS, not significant.

* Significant.

** Highly significant.

Environmental settings are quite different in native and introduced river systems. For example, although both are among the world's largest rivers, the Mississippi River flows southwards from Minnesota to the Gulf of Mexico, whereas the Yangtze River flows eastwards to the East China Sea. In addition, the extensive network of connected floodplain lakes characterizes the lower Yangtze basin, which is considered the primary natural living and growing habitat of Chinese carps (Kolar et al., 2007), does not exist in North America. Thus, it is plausible that natural selection in a novel environment like the Mississippi River Basin has resulted in rapid population growth and range expansion.

Within the Yangtze River, the allozyme analyses detected no significant genetic difference between fish from the middle and lower stream in either bighead carp or silver carp (Zhao & Li, 1996). However, the significant genetic difference was revealed through mitochondrial coding gene and D‐loop sequence analyses (Lu et al., 1997). The morphological difference of bighead carp or silver carp in the middle and lower stream of the Yangtze River was found insignificant (Li et al., 1990). In North America, the study of phylogeography and populating genetics of introduced silver carp and bighead carp using microsatellite markers revealed very little population genetic structure, which is arguably consistent with rapidly spreading invasive species (Farrington, Edwards, Bartron, & Lance, 2017). However, the population genetic study of silver carp sampled from the original establishment core, invasion fronts and expansion areas using microsatellites and sequences of two mitochondrial genes and one nuclear gene intron detected significant genetic differences in eight of ten pairwise comparisons with samples collected from the MRB (Stepien, Snyder, & Elz, 2019).

Genetic diversity of bigheaded carps was found to be high in both native and invasive populations (Table 2) (Li et al., 1990, 2010, 2011). The analysis of allozyme loci showed the bigheaded carps in three native rivers (Yangtze, Pearl and Amur) possessed high genetic diversity compared to other fish species (Li et al., 1990). Compared with native populations, the introduced populations possessed lower haplotype diversity and nucleotide diversity (Li et al., 2010, 2011). The Yangtze silver carp possessed the highest genetic diversity. It is unexpected that the Amur bighead carp possessed the highest nucleotide diversity. One possible explanation is that the bighead carp in the Amur River had been introduced multiple times and from different locations such as Yangtze and Pearl (Li et al., 1990). Transportation of fish fry from the Perl River Basin and the Yangtze River Basin to other regions is a common practice (Li et al., 1990). A recent mtDNA study on bighead carp and silver carp in the Pearl River revealed the Pearl populations were dominated by genetic material from the Yangtze River, which calls for ceasing the stocking practice of Yangtze fingerlings in the Pearl River to preserve native genetic diversity (Li et al., 2020). The highest number of parsimonious informative sites was observed in bigheaded carps in the MRB, which suggests both carps might have experienced rapid evolution since introduction (Li et al., 2010, 2011).

**TABLE 2:** Genetic diversity of native and introduced/invasive populations in bighead carp and silver carp

Species	Population	Haplotype diversity (mtDNA)	Nucleotide diversity (mtDNA)	Nei's Gene diversity (H)	Shannon diversity (I)	*H_E_* (microsatellites)
Bighead carp	Yangtze	0.7870 ± 0.0308 (60)	0.0020 ± 0.0012 (60)	0.0659 ± 0.1333 (20)	0.1055 ± 0.1998 (20)	0.73 (290)
	Pearl	0.9143 ± 0.0559 (15)	0.0031 ± 0.0018 (15)	0.0510 ± 0.1155 (20)	0.0837 ± 0.1759 (20)	0.82 (21)
	Amur	0.9333 ± 0.0773 (10)	0.0035 ± 0.0021 (10)	0.0661 ± 0.1364 (20)	0.1051 ± 0.2024 (20)	
	Danube	0.7375 ± 0.0305 (57)	0.0014 ± 0.0009 (57)	0.0576 ± 0.1250 (10)	0.0933 ± 0.1876 (10)	
	MRB	0.6879 ± 0.0448 (48)	0.0043 ± 0.0023 (48)	0.0540 ± 0.1221 (20)	0.0875 ± 0.1832 (20)	0.64 (452)
Silver carp	Yangtze	0.9515 ± 0.0141 (58)	0.0094 ± 0.0047 (58)	0.0314 ± 0.1033 (20)	0.0480 ± 0.1537 (20)	0.72 (18)
	Pearl	0.8571 ± 0.1023 (7)	0.0076 ± 0.0044 (7)	0.0529 ± 0.1236 (20)	0.0832 ± 0.1869 (20)	0.77 (17)
	Amur	0.9253 ± 0.0230 (56)	0.0064 ± 0.0033 (56)	0.0233 ± 0.0883 (20)	0.0373 ± 0.1304 (20)	0.72 (21)
	Danube	0.8431 ± 0.0447 (26)	0.0056 ± 0.0029 (26)	0.0122 ± 0.0619 (20)	0.0194 ± 0.0950 (20)	
	MRB	0.7351 ± 0.0361 (94)	0.0030 ± 0.0016 (94)	0.0134 ± 0.0614 (18)	0.0222 ± 0.0964 (18)	0.66 (454)

Note

Data from (Farrington et al., 2017; Li et al., 2010, 2011; Yan et al., 2010, 2011)

Analysis of RAD‐Seq sequences showed nucleotide diversity and heterozygosity values that were much higher in the Yangtze than the Illinois and Mississippi rivers (*unpublished*). Using microsatellite markers, Farrington et al. (2017) found moderately low genetic diversity in North American populations of bighead and silver carps but remained high as compared to other species of fishes. Population genetic study of silver carp revealed moderate levels of genetic diversity, with more mtDNA haplotypes and unique microsatellite alleles in the established core area (Stepien et al., 2019). We recently sequenced the draft genome of invasive bighead and silver carps and estimated heterozygosity of 0.0021 in bighead carp and 0.0036 in silver carp (Wang, Gaughan, et al., 2019). Heterozygosity in bigheaded carps ranged from moderate to high levels when compared with 10 other fin‐rayed species (Wang, Gaughan, et al., 2019). Genomic heterozygosity levels in native populations of bigheaded carps remain unknown.

## 4 EXTENSIVE HYBRIDIZATION OF BIGHEADED CARPS IN THE UNITED STATES

Hybridization and introgression are pervasive evolutionary events among animal and plant taxa worldwide (Mallet, 2005; Schwenk, Brede, & Streit, 2008). Bighead and silver carps have similar in morphology, reproductive behavior, and karyotypes (de Almeida‐Toledo, Bigoni, Bernardino, & de Almeida Toledo Filho, 1995). The two species can be hybridized artificially; however, natural hybridization between the two carps is believed to occur very rarely in their native ranges despite the significant overlap in spawning times and locations (Kolar et al., 2007; Lamer et al., 2010; Yu et al., 1988). On the contrary, it is relatively common in invasive areas in the Mississippi River Basin (Lamer et al., 2015). Hybrids between the two carps in the MRB were first detected morphologically in the Missouri River in around 2005 and molecularly in the Mississippi and Illinois rivers in 2006 (Lamer et al., 2010). In our mtDNA analysis of bigheaded carps sampled from the MRB during 2006–2007, we detected one hybrid out of 49 morphologically identified bighead specimens and 12 hybrids out of 106 silver carp specimens. Twisted gill rakers occurred in some hybrids (likely F1) in the MRB (Figure 2). However, the gill rakers are unreliable in the detection of post‐F1 hybrids. The analysis of diagnosis SNP markers identified 44% samples of bigheaded carps showed a strong maternal influence of silver carp that drives hybridization (Lamer et al., 2015). Given that pure bighead and silver carps will become increasingly less common, a hybrid swarm may result from the continuing introgression between these species (Lamer et al., 2015). A recent survey indicates early generation hybrids, although rare, may be associated with range expansion of invasive bigheaded carps. The underlying mechanisms such as a genetic combination that could result in more rapid adaptation to range expansion, however, require further genomic studies (Coulter, Brey, Lamer, Whitledge, & Garvey, 2019).

We conducted a transcriptomic analysis of pure and hybrid bigheaded carps and found one of four hybrids studied had a significantly lower number of transcripts in important biological processes, indicating a potential loss of fitness in F1 hybrids (Wang, Lamer, & Gaughan, 2016). The transcriptomic comparison of pure and hybrid bighead carps allowed confident detection of introgression and hybridization history (Wang, Lamer et al., 2016). We also sequenced the genomes of bighead and silver carps and their hybrids and found a high genomic similarity (over 96% in all syntenic blocks) between bighead and silver carps, indicating their genomic compatibility, a prerequisite for interspecific hybridization (Wang, Gaughan, et al., 2019). We mapped the sequences of two F1 hybrids and the majority of SNPs in F1 hybrids appeared to have no harmful effects on the individuals (Wang, Gaughan, et al., 2019). Bigheaded carps are invasive species, and live fishes are prohibited from being transported or possessed in the United States; we thus conducted the cross experiment in China. We observed a high fertilization rate between bighead and silver carps and high embryonic viability of F1 hybrids and found no significant difference between pure and hybrid crosses (Wang, Gaughan, et al., 2019). Our findings of high genomic similarity, along with the possible absence of reproductive isolation conditions in introduced environments, might have resulted in early hybridization of introduced bigheaded carps that escaped confinement and entered the MRB (Wang, Lamer, et al., 2016; Wang, Gaughan, et al., 2019). Preadapted genomic features such as positive selection genes associated with environmental adaptation and other invasion‐related traits in bigheaded carps and postintroduction hybridization between them collectively contribute to their successful invasions in the MRB (Wang, Gaughan, et al., 2019).

## 5 DRAMATIC DECREASE OF NATURAL RESOURCES IN NATIVE RIVERS

The fishery resources of bigheaded carps have decreased in the Yangtze, Pearl, and Amur Rivers during the past several decades (Chen, Xiong, Wang, & Chang, 2009; Wu & Wang, 1992; Yu et al., 1988). In the 1980s, the catch production of marketable‐sized fishes was about half that of the 1950s whereas the catch number of natural fry was only a quarter of the 1960s (Li, 1996; Li et al., 1990). Surveyed changes of larval Chinese carp abundances in the middle reach of the Yangtze River demonstrate the construction of the Three Gorges Dam (TGD) near the Yichang section between the upper and middle reaches apparently had a severe impact (Duan et al., 2009; Zhang et al., 2012). Bigheaded carps require longer river reaches for adult fish to migrate and spawn and demand fast water currents to transport fertilized eggs and larvae. It thus is important to consider the ecological consequences of dams in regulating policies related to their discharges (Hu et al., 2015; Wang, Kao, et al., 2019).

In addition to hydroelectric projects, other factors such as overfishing, species invasion, and water pollution could have contributed to population decline. For instance, Li and Wilcove (2005) found that overexploitation threatened 78% of the species listed in the IUCN Red Book, followed by habitat destruction (70%), pollution (20%), exotic species (3%) and diseases (<1%). A survey by Quan & Shen (2004) showed that the Yangtze estuary and its adjacent waters have become severely polluted. A report by the Ministry of Environmental Protection of China described that Amur sturgeon (*Acipenser schrenckii*), tench (*Tinca tinca*), channel catfish (*Ictalurus punctatus*), and other exotic fish species had invaded the upper Yangtze (The Ministry of Environmental Protection the People’s Republic of China, 2008). To protect natural resources of bigheaded carps in native rivers, several policies have been implemented by state and provincial governments in China, including stocking of bighead carp and silver carp fingerlings, the prohibition of fishing, development of natural reserves, ecological rehabilitation and fishery management (Chapman et al., 2016; Chen, 2018; Qiao, Liao, Cai, & Xu, 2014; Shi, Liu, Zhang, & Xu, 2005; Zhong, Wu, & Lian, 2007). These policies do not seem to be sufficient for the recovery of wild populations of bigheaded carps (Chapman et al., 2016). The Chinese government has announced a policy for a 10‐year fishing ban in the Yangtze River starting from January 1, 2021 (Liu, Peng‐Cheng, & Li, 2019).

## 6 EXPONENTIAL INCREASE OF WILD POPULATIONS IN THE MRB

Bigheaded carps were introduced into the southern United States to improve water quality in the fish culture ponds in the early 1970s (Kolar et al., 2007). The carps were found in the wild in the 1980s and have been expanding their territories since. Potential impacts of introduced bigheaded carps include predation on plankton populations, which could lead to negative effects on native fishes and invertebrates (Fuller et al., 1999; Irons et al., 2007). Reduced condition factor of two native fish species (gizzard shad and bigmouth buffalo) was found coincident with invasion of non‐native bigheaded carps in the Illinois River (Chick & Pegg, 2001), suggesting the competition between invasive carps might have resulted in reduced fitness in native species (Irons et al., 2007). In addition, silver carp tend to leap out of the water when disturbed (Schofield, Williams, Nico, Fuller, & Thomas, 2005). Jumping silver carp have caused some personal injuries and property damage to recreational boaters and anglers in the Midwest USA (Kolar et al. 2007). It has been concern that bigheaded carps may colonize the Great Lakes and eventually establish self‐reproducing populations (Cooke & Hill, 2010). The Great Lakes will likely experience ecosystem degradation and fisheries declines (Chapman et al., 2016).

To prevent the introduction and establishment of Asian carps in the Great Lakes, the Asian Carp Regional Coordinating Committee has been developing Annual Action Plans since 2010 that contain a series of detection, perversion and control projects for a comprehensive, multi‐pronged, science‐based management strategy (Asian Carp Regional Coordinating Committee, 2020). According to the interim summary report by the Asian Carp Monitoring and Response Plan, a total of 374,288 fish representing 73 species and six hybrid groups were sampled from 2010–2017 (The Monitoring & Response Workgroup, 2017). Asian carps (bighead carp, silver carp, and grass carp) were found to contribute 83.4% of the catch while others along with common carp made up an additional 16.6% in the Dresden Island, Marseilles and Starved Rock pools of the Upper Mississippi River System (The Monitoring & Response Workgroup,^2017^). The Long Term Resource Monitoring (LTRM) element of the US Army Corps of Engineers Upper Mississippi River Restoration Program represents one of the world's largest river‐related monitoring and surveying effort for over 20 years (Ratcliff, Gittinger, O’Hara, & Ickes, 2014). Fish abundance (catch per unit effort from electrofishing) collected from 1994 to 2013 through LTRM showed empirical evidence of a negative effect of invasive silver carp on native sport fish (Chick, Gibson‐Reinemer, Soeken‐Gittinger, & Casper, 2019). Harvest appears to be the only effective method currently being practiced as a control for existing populations of bigheaded carps (Chapman et al., 2016). One issue associated with this harvest strategy is the potential for rapid genetic change in bigheaded carps (Chapman et al., 2016). In addition, the harvest may enhance movements or growth of remaining individuals (Coulter, MacNamara, Glover, & Garvey, 2018). Alternative tools other than the barriers and harvest such as acoustic deterrents, carbon dioxide, microparticles, and barge entrainment are under research and development (Asian Carp Regional Coordinating Committee, 2020).

## 7 DISCUSSION

### 7.1 Evolutionary history of bigheaded carps

Much progress has been made through evolutionary studies of bigheaded carps, particularly with molecular data. It appears existing bigheaded carps originated from the Yangtze‐Huanghe Rivers and two lineages of each species occurred in the Yangtze River populations. The latter hypothesis could be further tested using more robust tools such as RAD‐Seq (Davey & Blaxter, 2010) or whole‐genome resequencing (Huang et al., 2009) and by analyzing other widely distributed species such as *Luciobrama microcep*halus, *Ochetobius elongates*, *Elopichthys bambusa* (Liu et al., 2019) in the Yangtze River. Although there is a discrepancy in the estimated divergence time between bighead and silver, both bighead and silver carps might have existed in the Pliocene Epoch (Li, 1981). Regarding the evolution of bigheaded carps, several questions remain to be answered, including whether they evolved through sympatric speciation, allopatric speciation or both, a case similar to lake whitefish (Lu, Basley, & Bernatchez, 2001). It is unknown whether bighead carp or silver carp is an ancestral species in *Hypophthalmichthys*. With the genus *Hypophthalmichthys*, there is another species, large‐scale silver carp *H. harmandi*, which is native to Hainan, China and Vietnam (Froese & Pauly, 2000). It is plausible large‐scale silver carp evolved through peripatric speciation from silver carp because large‐scale silver carp has a narrow distribution and overlaps only partially with the southern range of silver carp distribution. Molecular data could allow the detection of contact zones and the inference of their evolutionary history.

### 7.2 Interspecific hybridization and hybrid swarm

Silver carp and bighead carp are known to hybridize and produce viable offspring under artificial manipulations (Kolar et al., 2007). Hybridization between the two species has not been reported from wild populations in their native waters (Yi, Wahab, & Diana, 2006). However, hybrids are pervasive throughout the MRB (Lamer et al., 2015; Lamer, Ruebush, McClelland, Epifanio, & Sass, 2019). These are not likely to be the result of artificially introduced hybrids because neither silver carp nor hybrids are used in U.S. aquaculture (D. Chapman, *personal communication*). It appears certain the introgression of bigheaded carps has resulted in a hybrid swarm, although a clear understanding of population dynamics of parental and hybrid species is lacking (Lamer et al., 2015). The F_1_ hybrids with twisted gill rakers may lead to reduced fitness in food competition, which can be explained by the low percentage of F1 individuals found throughput the MRB (Lamer et al., 2015; Wang, Gaughan, et al., 2019). The reproductive potential was found with no difference between hybrids and parental species, suggesting the low frequency of early generation hybrids may have resulted from poor condition factors, low postreproductive survival, or selection pressures acting on juvenile or immature life stages (Lamer et al., 2019). Early generation hybrids are suspected to play a role in driving range expansion of two invasive fishes (Coulter et al., 2019). A long‐term demographic survey of pure and hybrid bigheaded carps in conjunction with genomic studies may unveil the mechanisms and evolutionary consequences of hybridization and introgression in bigheaded carps.

Several studies suggest that a combination of genetic and environmental factors may contribute to the success of bighead and silver carp hybrids in the Mississippi River Basin (Lamer et al., 2019; Wang, Lamer, et al., 2016; Wang, Gaughan, et al., 2019). Further investigations using tracking systems such as a stationary acoustic telemetry array are needed to identify environmental factors that may drive the hybridization of bigheaded carp in the MRB (Coulter, Brey, et al., 2018). Comparative genomic and transcriptomic studies will allow disentangling the genetic mechanisms that underlie potential hybrid inferiority and hybrid vigor (Wang, Lamer, et al., 2016; Wang, Gaughan, et al., 2019). Providing the construction of the world‐largest hydroelectric facility in the Yangtze River has led to a more homogenous environment in the Three Gorges reservoir, we suspect bigheaded carps may change their reproductive ecology and/or behavior that facilitate interspecific hybridization, a situation currently occurring in the MRB. Nevertheless, natural hybridization between two introduced species that has resulted in a hybrid swarm in many rivers and lakes of the MRB offers a unique opportunity to study hybrid speciation, ecological adaptation, eco‐evolutionary dynamics and more (Pelletier, Garant, & Hendry, 2009).

### 7.3 Native resource conservation and invasive species control

In China and some other countries, bighead and silver carps are excellent food fish. Silver and bighead carps ranked respectively second and fifth in world aquaculture fish production in 2016 (FAO, 2018). On the contrary, natural resources of bigheaded carps within their native ranges have declined radically since the late 1950s for many reasons such as overfishing, hydroelectric facility construction (e.g., damming), and aquatic environmental pollution (Duan et al., 2009; Liu et al., 2019). With the increase of aquaculture and significant decline of natural resources, the populations of bigheaded carps in their native ranges have experienced changes in morphological and life‐history traits during the past half‐century, including genetic diversity (Li et al., 2010, 2011; Yu, Tang, & Li, 2010). This raises a special concern that the loss of genetic diversity could result in the degradation of phenotypic traits, such as rapid growth rate and high fecundity. On the contrary, bigheaded carps were introduced to North America for water quality improvement and aquaculture production enhancement (Kolar et al., 2007). Bigheaded carps are overpopulated biomass and dominated in terms of biomass in many regions of the MRB (The Monitoring & Response Workgroup, 2017). Unexpectedly, introduced bigheaded carps can hybridize in the MRB and a hybrid swarm is under evolving (Lamer et al., 2010, 2015; Wang, Kao, et al., 2019). To address issues related to the management and control of bigheaded carps in the Mississippi River and the resources restoration and genetic diversity preservation in the Yangtze River, governmental agencies and research scientists from the United States and China have been working collaboratively and diligently with many fruitful outcomes (Chapman et al., 2016). A formal consortium shall assist in achieving the long‐term mission set by the two countries for biodiversity protection, which eventually benefits the whole world.

## CONFLICT OF INTEREST

None declared.

## Data Availability

The NCBI BioProject accession numbers for genomic sequences are PRJNA305140 and PRJNA305141. The mtDNA genomic data can be accessed with the NCBI accession numbers NC_010194 and NC_010156 for bighead carp and silver carp, respectively.
